# *Tasselseed5* overexpresses a wound-inducible enzyme, *ZmCYP94B1*, that affects jasmonate catabolism, sex determination, and plant architecture in maize

**DOI:** 10.1038/s42003-019-0354-1

**Published:** 2019-03-25

**Authors:** China Lunde, Athen Kimberlin, Samuel Leiboff, Abraham J. Koo, Sarah Hake

**Affiliations:** 1 University of California, Berkeley, CA 94720 USA; 2 Plant Gene Expression Center, U.S. Department of Agriculture-Agricultural Research Service, 800 Buchanan Street, Albany, CA 94710 USA; 3 Department of Biochemistry, University of Missouri, Columbia, MO 65211 USA; 4 Interdisciplinary Plant Group, University of Missouri, Columbia, MO 65211 USA

## Abstract

Maize is monecious, with separate male and female inflorescences. Maize flowers are initially bisexual but achieve separate sexual identities through organ arrest. Loss-of-function mutants in the jasmonic acid (JA) pathway have only female flowers due to failure to abort silks in the tassel. *Tasselseed5* (*Ts5*) shares this phenotype but is dominant. Positional cloning and transcriptomics of tassels identified an ectopically expressed gene in the CYP94B subfamily, *Ts5 (ZmCYP94B1)*. CYP94B enzymes are wound inducible and inactivate bioactive jasmonoyl-L-isoleucine (JA-Ile). Consistent with this result, tassels and wounded leaves of *Ts5* mutants displayed lower JA and JA-lle precursors and higher 12OH-JA-lle product than the wild type. Furthermore, many wounding and jasmonate pathway genes were differentially expressed in *Ts5* tassels. We propose that the *Ts5* phenotype results from the interruption of JA signaling during sexual differentiation via the upregulation of *ZmCYP94B1* and that its proper expression maintains maize monoecy.

## Abstract (web summary)

China Lunde et al. used cloning, transcriptomics, and metabolomics to identify the *Tasselseed5* (*Ts5*) gene in maize, and its role in the jasmonate metabolism. They find that the phenotype of the *Ts5* mutants is the result of interrupted jasmonic acid signaling during sexual differentiation.

## Introduction

Most plants produce hermaphroditic flowers with the male organs, stamens, surrounding an inner whorl of female organs, the pistils. Fertilization occurs when pollen from the stamens successfully reaches the ovule within the pistil. While this arrangement assures successful seed production, it may also lead to inbreeding and reduced fitness. Plants have evolved several mechanisms to avoid inbreeding. One mechanism is genetic self-incompatibility, in which distinct alleles in the male pollen and female pistil are required for successful seed set^1^. Another mechanism is physical separation, which involves the development of unisexual flowers. In monoecy, male and female flowers are on the same plant^2^. It is estimated that 30% of the plant species produce some unisexual flowers^3^, including maize. In contrast, diecious plants, such as *Cannabis*, *Corylus*, or *Asparagus*, have separate male and female plants.

Maize produces separate staminate (male) and pistillate (female) inflorescences called the tassel and the ear, respectively. All grasses contain flowers (referred to as florets) flanked by sterile bracts called glumes within spikelets, units of the grass inflorescence. During early floral development, the two florets in both the tassel and the ear spikelets are hermaphroditic, and monoecy is conferred by the selective abortion of pistillate organs in tassel florets and the arrest of staminate organs in the ear floret^4,5^.

A large number of sex-determination mutants have been identified in maize, given the easy visibility of *tasselseed* mutants in which silks (pistils) and kernels (seeds) are found in the normally male tassel. Recessive mutants, *ts1* and *ts2*, and dominant mutants, *Ts3* and *Ts5*, fail to abort carpels in tassels and also fail to abort the lower floret in ears^6–8^. *ts1* encodes a lipoxygenase, also called *ZmLox8*, that acts in jasmonic acid (JA) biosynthesis^9,10^, and *ts2* encodes a monocot-specific^3^ short-chain alcohol dehydrogenase^11^. Both *ts1* and *ts2* have a reduced plant height^8^. Another *tasselseed* mutant was created by knocking out the duplicated orthologs of *OPR3*, a major *OPR* (12-oxo-phytodienoic acid reductase) gene in *Arabidopsis* that acts in JA biosynthesis^12,13^. The resulting maize *opr7opr8* mutants are phenotypically similar to *ts1* and *ts2* mutants^14,15^.

Other mutants with silks in the tassels include *ts4*, which encodes an miR172 microRNA, and *Ts6*, which has a mutation in the *ts4*-binding site^16^. Brassinosteroid biosynthetic mutants^17,18^ and epigenetic mutants such as *required to maintain repression6 (rmr6)*^19^ also show feminized tassels. Clearly, many modes of regulation are necessary to keep hormone action balanced for proper sex determination^20^.

Here, we use positional cloning, transcriptomics, and metabolomics to identify the *Ts5* gene and its role in jasmonate metabolism. The mechanism of *Ts5* function reveals a role for jasmonate catabolism through the ω-oxidation pathway in both attenuating response to wounding and specification of sexual identity.

## Results

### *Ts5* tassels are feminized and ear florets fail to abort

Maize bears a terminal staminate inflorescence (tassel) and lateral pistillate inflorescences (ears), which arise in the axils of leaves. Early development of ear and tassel is similar except that the tassel initiates branches prior to initiating spikelets. Both male and female spikelets initiate two floral meristems inside the sterile glumes. In the tassel spikelet, the pistils abort producing two male florets. In the ear, the stamens arrest and the pistil of the lower floret aborts, leaving a single pistil to grow out and receive pollen in the female spikelet^5^.

**Fig. 1:**
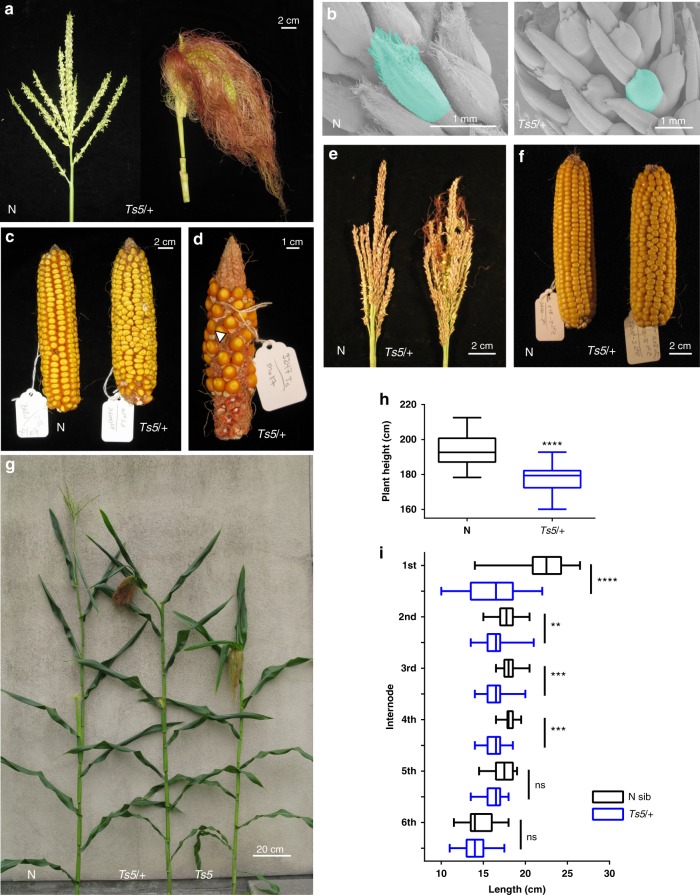
Inflorescence and vegetative phenotype of *Ts5*. **a** Normal and *Ts5/* *+* mature tassels in Mo17. **b** SEM micrograph of central spike of 4 cm tassel of normal and *Ts5/* *+* in Mo17. **c** Normal and *Ts5/* *+* ears in Mo17. **d** A poorly pollinated *Ts5/* *+* ear in Mo17 showing empty pericarps. Arrow: appressed empty pericarp of the lower floret. **e** Normal and *Ts5/* *+* mature tassels in B73. **f** Normal and *Ts5/* *+* ears in B73. **g** Mature plants of normal, *Ts5/* *+* , and *Ts5/Ts5* in A188. **h** Plant height (cm) of normal siblings (*n* = 30) and Ts5/ + (*n* = 31) in A188. Height was significantly different by two-tailed unpaired *t* test, *P* < 0.0001 (*t* = 6.778, df = 59; 95% CI −20.21 to −11.00). Bar, mean; whiskers, SD. **i** Graph of upper internode lengths of the same plants graphed in panel (**h**), normal siblings (*n* = 30) and *Ts5*/ + (*n* = 31) in A188. The entry 1^st^ internode is measured from the last tassel branch to the first subtending node. Means of the first four internodes measured were significantly different, one-way ANOVA with Sidak’s multiple comparison test adjusted for multiple comparisons: 1^st^, *t* = 13.96, *P* < 0.0001, 95% CI = 4.778–7.0137; 2^nd^, *t* = 3.3874, 95% CI = 0.3131771–2.548113, *P* = 0.0047; 3^rd^, *t* = 4.162688, 95% CI = 0.6405964–2.875533, *t* = 4.287441, 95% CI = 0.6932846–2.928221, *P* = 0.0002; 4^th^, *P* = 0.0001, global DF = 353, ns = non-significant. Bar, mean; whiskers, SD. SEM, scanning electron microscope

*Ts5* tassels are feminized; the pistil fails to abort and, consequently, stamen development is arrested in affected florets. Although covered in silks, the *Ts5* tassel remains branched (Fig. 1a–e) as previously described^6–8^. Scanning electron micrographs reveal that in developing tassels of *Ts5/+* , glumes are short and glabrous (Fig. 1b), resembling those of ear glumes, a phenotype shared with *ts2*^11^, and indicating that the entire spikelet is feminized in the Mo17 background. In contrast, tassel glumes of normal siblings are long and produce trichomes (Fig. 1b). *Ts5/* *+* ears are also abnormal; they fail to abort the lower floret, leading to disordered vertical kernel files (Fig. 1c–f)^6,7^. In cases of reduced seed set, unfilled pericarps are visible, appressed to the kernel of the upper floret (Fig. 1d); these are present but hidden by filled kernels in fully pollinated ears as in Fig. 1c, f.

In crossing *Ts5* to different inbred backgrounds, we observed strong differences in expressivity. In the Mo17 background, the tassel is completely feminized and produces no pollen (Fig. 1a). In B73 tassels, *Ts5* displays a mild phenotype and is male fertile (Fig. 1e). Spikelets in B73 *Ts5* tassels are a mix of either male or female identity, but female florets can be found inside male glumes. The weak expressivity seen in B73 tassels, however, is not true for the ear because lower floret abortion still fails and the rows are uneven (Fig. 1f). The phenotype of *Ts5* in A188 is intermediate between that of Mo17 and B73. The tassel is highly feminized, but still produces pollen. We quantified tassel-feminization traits by measuring two ratios, the number of feminized branches/total branch number (FBN/TBN), and the length of the main spike that was feminized/spike length (FSL/SL) and found that feminization increases with increase in copies of *Ts5*. In A188, homozygotes have more feminization along the main rachis and heterozygotes have an intermediate phenotype (Supplementary Fig. 1). Raw data used to create each graph are available in Supplementary Data 4.

*Ts5* plants in A188 are noticeably shorter. Plants heterozygous for the dominant *Ts5* allele have intermediate heights and *Ts5* homozygotes are the shortest (Fig. 1g). We quantified height differences between *Ts5*/ + and wild type (A188, BC4) and found that mutants were nearly 16 cm shorter (Fig. 1h). Using these same individuals, we measured the internode length to determine the cause of the height difference and found a reduction in elongation of the four internodes immediately subtending the tassel (Fig. 1i). Genotype did not affect the lengths of the lower internodes.

### *Ts5* maps to *ZmCYP94B1* and has JA-deficient phenotypes

**Fig. 2:**
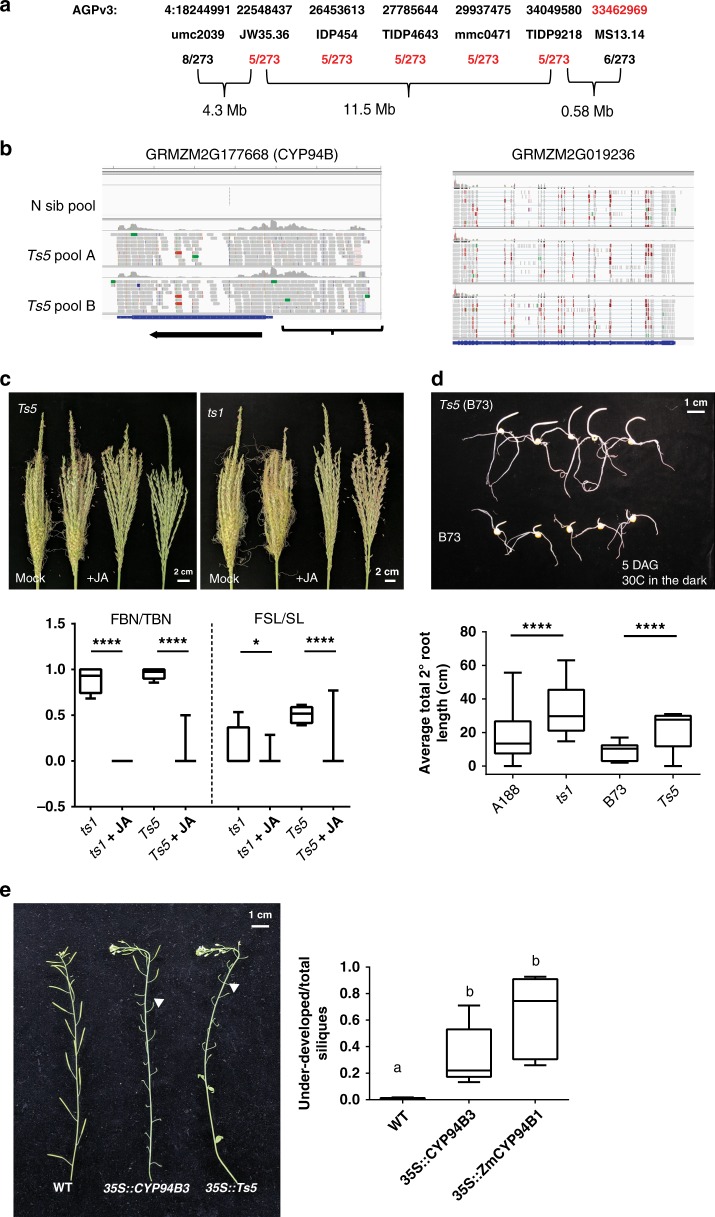
*Ts5* maps to a CYP94B that catabolizes JA, is corrected by JA application, and causes under-developed siliques when overexpressed in *Arabidopsis*. **a** Schematic of the *Ts5* mapping locus. Red text indicates loci lacking recombination or marker order inconsistent with B73 AGPv3. **b** A CYP94B (GRMZM2G177668) is upregulated in *Ts5/* + tassels relative to normal siblings in the Mo17 background visualized in IGV. Ectopic 5′ reads are indicated by brackets. A linked gene (GRMZM2G019236) has similar read counts in each pool. **c** Mature tassel phenotypes after the exogenous application of JA to developing tassels. Graphs of the ratios of feminized branch number to total branch number (FBN/TBN) and feminized spike length to total spike length (FSL/SL) of *ts1* (*n* = 11), *ts1* *+* JA (*n* = 8), *Ts5* (*n* = 12), and *Ts5* *+* JA (*n* = 11). Bar, mean, whiskers, SD. Treated mean of FBN/TBN and FSL/SL for *ts1* and *Ts5* are significantly less than untreated using an unpaired, one-tailed Student’s *t* test with Welch’s correction, *ts1* FBN/TBN treated vs untreated *t* = 24.0708, df = 10.0000, *P* *<* 0.0001, 95% CI = −0.980591 to −0.814434; *ts1* FSL/SL treated vs untreated *t* = 1.799 df = 15.18, *P* = 0.0459, 95% CI = −0.2855 to 0.02397; *Ts5* FBN/TBN treated vs untreated, *t* = 18.8039, df = 12.4881, *P* < 0.0001, 95% CI = −1.01137 to −0.802148, *Ts5* FSL/SL treated vs untreated,* t* = 5.94347, df = 12.3037, *P* < 0.0001, 95% CI = −0.600572 to −0.279006. **d** Photo of seedling phenotypes after growing in the dark for 5 days (30 °C) and graph of mean total secondary root length of *ts1* in the A188 background (*n* = 12), A188 (*n* = 10), *Ts5* homozygotes in the B73 background (*n* = 12), and B73 (*n* = 11). Mean root lengths of *ts1* and *Ts5* were significantly longer than those of wild type by two-tailed unpaired Student’s *t* test: for *ts1*, *P* = 0.0419, *t* = 2.173557, df = 20, 95% CI = 0.6021773 to 29.28449; for *Ts5*, *P* = 0.0036, *t* = 3.272358, df = 21, 95% CI = 4.680919 to 21.00393. **e** Photo of mature *Arabidopsis* lines *Col-O*, *CYP94B3-OE*, and *ZmCYP94B1-OE* showing under-developed siliques in both overexpression lines. Graph of the mean ratio of under-developed to normal siliques in each line is shown. Letters denote that wild-type plants are significantly distinct using two-tailed Student’s *t* test with Welch’s correction: WT vs 35S::CYP94B3, *t* = 3.123, *P* = 0.0353, 95% CI = 0.03581 –0.6044, df = 4.009; WT vs 35S::ZmCYP94B1, *t* = 4.538, *P* = 0.0105, 95% CI = 0.2446 –1.015, df = 4.005; 35S::CYP94B3 vs 35S::ZmCYP94B1, *P* = 0.1136. Bar, mean; whiskers, SD

*Ts5* appears on a genetic linkage map published by R.A. Emerson^21^. Current genetic mapping data placed the *Ts5* mutation within bin 4.03 (www.maizegdb.org)^22^. Using molecular markers, we found a lack of recombination between umc2039 and five adjacent markers. In our mapping population, TIDP9218 was distal to MS13.14, compared to its reported location in B73 AGPv3. We fine-mapped *Ts5* to a 15 Mb interval containing 65 genes between umc2039 and our custom indel marker JW35.36 (Fig. 2a; for primer sequences, see Supplementary Data 1).

To identify the potential mutations within these genes, we performed RNA sequencing (RNA-Seq) using 9–11 mm developing tassels from *Ts5/* *+* and normal siblings in the phenotypically expressive Mo17 background. This approach enriched for potential transcripts causal for the phenotype and excluded secondary transcripts simply related to ectopic silk production. Plants were genotyped by polymerase chain reaction (PCR) using flanking markers. A *CYP94B* gene GRMZM2G177668 was not expressed in the normal sibling pool but was highly expressed in two *Ts5/* *+* pools when comparing mapped reads (logFC 11.69) (Fig. 2b; Supplementary Data 2). In addition to an increase in read number, ectopic 5′ reads were detected, mapping to an area annotated as being 5′ of the canonical transcriptional start site of GRMZM2G177668. These 5′ reads are unique to *Ts5* and were not found in other published transcriptomes^10,23,24^, suggesting that the *Ts5* phenotype is the result of a novel transcript at this locus. Because *Ts5* behaves as a dominant mutation, the expression of a novel transcript or the overexpression of a normal transcript could be causal. The sequence reads of adjacent genes were similar in the *Ts5/* *+* pools compared to the normal pool (Fig. 2b), implying that although the order of two markers (MS13.14 and TIDP9218) is reversed in the mapping population, a gross rearrangement affecting linked genes had not occurred. Two other genes within the mapping interval were slightly upregulated in *Ts5* mutant pools. GRMZM2G079452 (logFC 1.35) is annotated as a hypothetical protein whereas GRMZM2G112795 (logFC 1.07) is annotated as a putative uncharacterized protein. Otherwise, the expression of nearby genes was unchanged between *Ts5* and normal plants, suggesting that the chromosomal rearrangement did not broadly impact regional transcription, and that the increase in GRMZM2G177668 expression is most likely the specific cause of *Ts5* phenotypes.

We sought to confirm that *Ts5* is in the JA pathway given that GRMZM2G177668 encodes a paralog of an enzyme known to catabolize bioactive jasmonate^25,26^ and *Ts5* shares phenotypes with jasmonic acid biosynthetic mutants^14,15^. We tested if an exogenous JA application could block the growth of silks in homozygous *Ts5* mutant tassels and suppress the mutant phenotype (Fig. 2c). We used *tasselseed1 (ts1*), a mutation in a JA biosynthetic enzyme, as a control. The feminization of that mutant was previously shown to be reversed by the exogenous application of JA^9^. JA or a mock solution was applied directly into the whorls of 4-week-old plants every 2 days for 2 weeks at a concentration of 1 mM as they transitioned from vegetative to inflorescence development. FBN/TBN and FSL/SL were quantified and compared to the reduction, after treatment, of *ts1* mutants (Fig. 2c). *Ts5* tassels responded similarly to *ts1* tassels, revealing that high concentrations of exogenous JA can rescue the feminization of the *Ts5* phenotype. We applied JA at a concentration of 1 mM, but endogenous levels are much lower. This finding is consistent with *Ts5* encoding an enzyme that catabolizes JA, though it is unable to metabolize an excess of JA applied directly to the developing tassel. Similar cases have been reported with transgenic lines overexpressing enzymes in the JA catabolic pathways^26–28^.

Since *Ts5* could be corrected by the addition of JA, we measured root traits, known to be affected in other JA biosynthetic mutants such as *opr7opr8* in maize^14^ or allene oxide cyclase mutants in rice^29^. In addition, the overexpression of either CYP94B1 or CYP94B3 in *Arabidopsis* has been shown to display a similar long root phenotype^26,27^. Dark-grown *Ts5* homozygous (produced after seven backcrosses to B73 (BC7)) seedlings have prematurely elongated coleoptiles and longer roots than B73 (Fig. 2d), similar to those found in *opr7opr8* double mutants. This suggests that *Ts5* plants have reduced bioactive jasmonate and that bioactive jasmonate negatively regulates root growth, as previously reported^14,15,29^. To assess the similarity in the function of *ZmCYP94B1* and *CYP94B3*, we overexpressed the maize gene in *Arabidopsis*. *ZmCYP94B1-OE* lines had a similar ratio of under-developed to developed siliques as *CYP94B3-OE* lines (Fig. 2e). *Arabidopsis* *CYP94B1-OE* lines have been reported to display similar flower-development defects as *CYP94B3-OE*^26^. From this experiment, we conclude that *ZmCYP94B1*, like *CYP94B1* or *CYP94B3*, functions to hydrolyze JA-Ile to control flower and fruit development in planta.

### *Ts5* mutants accumulate metabolites of jasmonate ω-oxidation

**Fig. 3:**
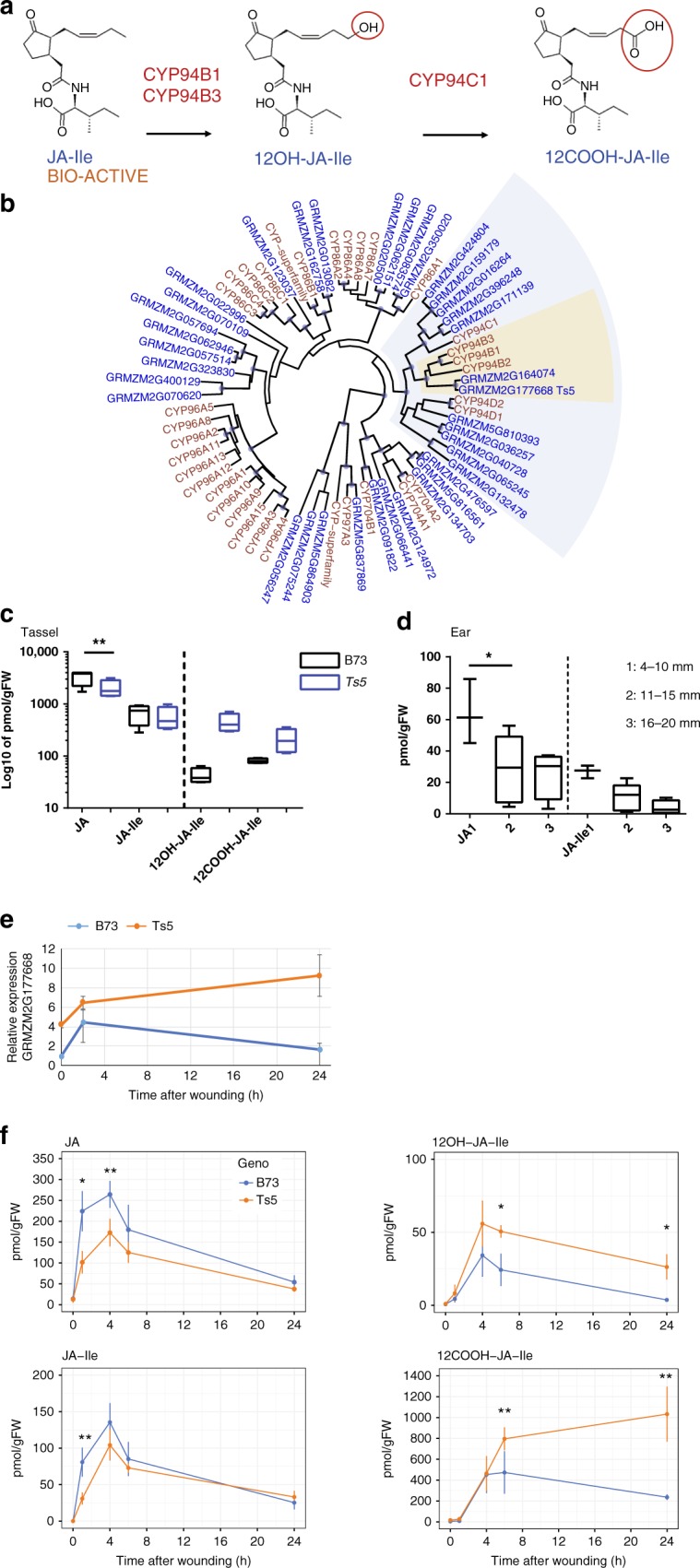
A wound-inducible CYP94B is upregulated in *Ts5*. **a** A diagram of steps of JA-Ile catabolism via the ω-oxidation pathway. **b** A phylogenetic tree of CYP genes from maize (blue) and *Arabidopsis* (brown). CYP94 genes (blue-gray) and CYP94B genes (yellow) are shaded. A blue dot at a node indicates branches with > 95% support. **c** Graph of jasmonate levels in developing tassels (2 cm) in B73 (black) and *Ts5* homozygotes (blue). The amount of 12OH-JA-Ile is significantly higher in *Ts5* than in B73 using a one-tailed Student’s *t* test with Welch’s correction, *P* = 0.0109, *t* = 4.348524 df = 3.037953, 95% CI = 112.0289 to 707.7894. **d** JA and JA-Ile levels in three sizes of B73 ears during early development. The amount of JA in 11–15 mm ears is significantly less than that in 4–10 mm ears by one-way ANOVA; *F* = 7.078, *P* = 0.008, DF = 18. Bar, mean; whiskers, SD. **e** Relative expression of GRMZM2G177668 measured by qRT-PCR at 0, 2, and 24 h after wounding in pooled (*n* = 4) second leaves of *Ts5* homozygotes (BC7 B73) and B73. Graph depicts means of three technical replicates. Error bars, SD. **f** Graphs depicting a time-course of LC–MS outputs of JA, JA-Ile, 12OH-JA-Ile, and 12COOH-JA-Ile accumulation (pmol/gFW) in wounded leaves of *Ts5* homozygotes (B73) and B73 at 0, 1, 4, 6, and 24 h. *Ts5* leaves had significantly less JA at 1 h (*t* = 4.567575, *P* = 0.0038, df = 6, 95% CI = −188.1638 to −56.88620) and 4 h (*t* = 3.548059, *P* = 0.0238, df = 4, 95% CI = −163.2358 to −19.91499) after wounding than B73. *Ts5* leaves had significantly less JA-Ile at 1 h (*t* = 4.802854, *P* = 0.0030, df = 6, 95% CI = −75.41736 to −24.50798) post-wounding than B73 leaves. *Ts5* leaves had significantly more 12OH-JA-Ile than B73 at 6 h (*t* = 3.961804, *P* = 0.0107, df = 5, 95% CI = 9.232525–43.35088) and at 24 h (*t* = 9.473349, *P* = 0.0002, df = 5, 95% CI = 18.97077–33.10019) post-wounding than B73 leaves. *Ts5* leaves had significantly more 12COOH-JA-Ile at 6 h (*t* = 5.007114, *P* = 0.0075, df = 4, 95% CI = 184.6060–644.1580) and at 24 h (*t* = 8.622493, *P* = 0.0003, df = 5, 95% CI = 626.7214–1159.130) post-wounding than B73 leaves. **f** Error bars, SD of four biological replicates. LC–MS, liquid chromatography–mass spectrometry

We profiled jasmonate metabolites in the developing tassels of *Ts5* homozygous plants (B73 BC7) and B73 wild-type controls using liquid chromatography–mass spectrometry to test whether they are predictably altered. *Arabidopsis* CYP94B1^26^ and CYP94B3^27^ are known to convert JA-Ile, the most bioactive form of jasmonate^30–33^ to the inactive form 12OH-JA-Ile during oxidative catabolism of JA^34,35^ as summarized in Fig. 3a. Comparison of CYP94B protein sequences from maize and *Arabidopsis* did not distinguish whether GRMZM2G177668 is more similar to *Arabidopsis* CYP94B3 or to CYP94B1 (Fig. 3b). Both the *Arabidopsis* proteins, however, oxidize JA-Ile to 12OH-JA-Ile semi-redundantly leading to less-bioactive jasmonate^26,27,34,36^. Based on the phylogeny and evidence presented in this paper, we named GRMZM2G177668 *ZmCYP94B1*. Our phylogenic analysis also revealed that GRMZM2G164074 is a close paralog, although not differentially expressed in *Ts5* heterozygous tassels (Supplementary Data 2).

Compared to B73 wild type, steady-state levels of JA and JA-Ile are lower in *Ts5* tassels, whereas steady-state levels of 12OH-JA-Ile, the product of JA-Ile oxidation by *Arabidopsis CYP94B1* and *CYP94B3*, are higher in *Ts5* mutants (Fig. 3c). This result is consistent with the increased expression of GRMZM2G177668 promoting catalysis of active jasmonates in *Ts5* tassels. In *Arabidopsis*, the prolonged catabolism of JA-Ile provides more substrate for CYP94C1, which preferentially catalyzes carboxy-derivative formation, explaining the increased levels of its downstream product 12COOH-JA-Ile^34^, also seen in Fig. 3c. 12OH-JA is a wound-induced jasmonate, also known as tuberonic acid^37^, that results either from the hydrolytic cleavage of 12OH-JA-Ile by amidohydrolases^38,39^ or from the direct oxidation of JA^40,41^. We also profiled wild-type B73 ear jasmonates and found that the levels of all tested metabolites (JA, JA-Ile, 12OH-JA, 12OH-JA-Ile, and 12COOH-JA-Ile) were much lower in ears than in tassels. Moreover, the level of JA-Ile decreased significantly as wild-type ears grew from 6 mm (comprised of spikelet and spikelet pair meristems) to 20 mm (comprised of floral meristems) (Fig. 3d).

The CYP94B3 enzyme is known to be wound inducible in *Arabidopsis*^27,35^. To evaluate our hypothesis that *Ts5* is a maize functional homolog of *CYP94B*, we assayed its gene expression and profiled JA metabolites via a wounding time-course experiment. We found that GRMZM2G177668 is expressed at higher levels in *Ts5* homozygous (B73 BC7) leaves even before wounding (Fig. 3e), consistent with the increase seen in our RNA-Seq experiments in *Ts5* tassels (Fig. 2b). Post-wounding, GRMZM2G177668 levels increased within 2 h in both wild-type and *Ts5* plants. In wild type, GRMZM2G177668 expression levels dropped to pre-wounding levels within 24 h, yet GRMZM2G177668 expression levels remained high in *Ts5* (Fig. 3e). These results suggest that the regulation of the mutant allele is altered in *Ts5*, causing enhanced *ZmCYP94B1* transcript accumulation.

The JA metabolite profile in wounded *Ts5* homozygous leaves, in the B73 genetic background, is consistent with the prolonged and increased expression of *ZmCYP94B1*. JA-lle and JA levels were lower in *Ts5*, but 12OH-JA-lle and 12COOH-JA-lle levels are increased (Fig. 3f). Similar to reports in *Arabidopsis*^27^, the jasmonate catabolites showed a slight time lag behind the increase in JA and JA-Ile. One hour post-wounding, JA and JA-Ile had already increased by 30–60% of their highest levels, but 12OH-JA-Ile and 12COOH-JA-Ile had only increased by 5–10%. Importantly, JA and JA-Ile levels were lower in *Ts5* compared to B73 at–4-6 h post-wounding, whereas oxidized jasmonate levels were significantly higher in *Ts5*, consistent with the enhanced metabolism via the ω-oxidation pathway. Twenty-four hours post-wounding, the JA catabolites in wild-type plants, especially 12COOH-JA-Ile, were lowered to near starting levels, but remained high in the mutant. Taken together, these results support the hypothesis that *Ts5* is altered in JA metabolism due to the overexpression of GRMZM2G177668 because metabolites in the JA pathway are affected in *Ts5* mutants in a specific manner consistent with higher CYP94B enzymatic activity.

### *Ts5* differentially expresses JA signaling genes

Of the 231 significantly differentially expressed genes detected in our RNA-Seq analysis (false discovery rate (FDR) < 0.05), 89% were downregulated. Eight percent (11/131) of the annotated differentially expressed genes are involved in JA signaling (Supplementary Data 2; Supplementary Data 3), including lipoxygenase genes (*LOX1*, *LOX2*, and *LOX5*), an allene oxide synthase (*AOS* or *CYP74* *A*), an allene oxide cyclase (*AOC3*), and 12-oxophytodienoate reductase (*OPR1*). This finding is consistent with these JA-Ile-inducible genes being downregulated due to the increased turnover of JA-Ile in *Ts5* mutants. *ts2*, which encodes a dehydrogenase implicated in JA biosynthesis^11^, was also differentially expressed in *Ts5* tassels although we did not detect any difference in JA-Ile, nor several other jasmonates in *ts2* mutants compared to their non-mutant siblings following wounding (Supplementary Figure 2).

### *Ts5* interacts genetically with *ts2* and *sk1*

**Fig. 4:**
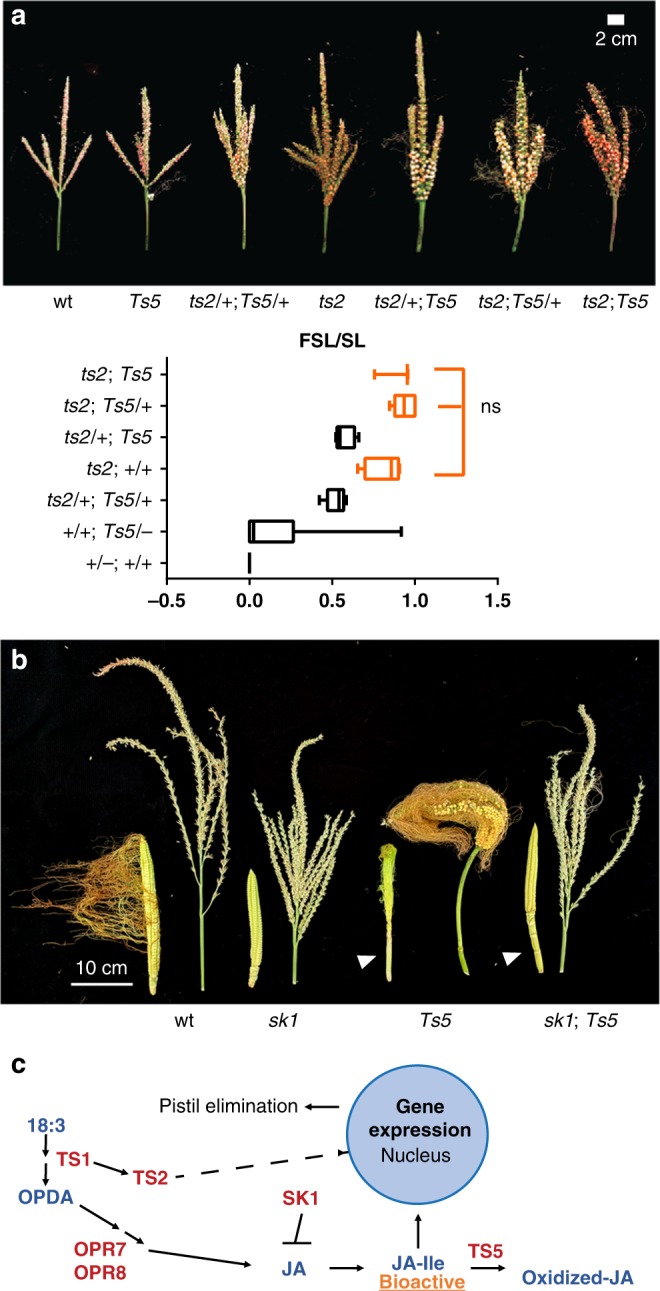
Genetic dissection of *Ts5*. **a** Analysis of *ts2;Ts5* double mutants in the A188 background and their effect on feminized spike length over spike length (FSL/SL). Photos are of mature tassels showing feminization, silks, and kernels in genotypes in which they are formed. In graph, measurements of individual plants (double homozygous mutants (*n* = 3), *ts2:Ts5/* *+* (*n* = 6), *ts2/* *+* ;*Ts5* (*n* = 5), *ts2:* *+* */* *+* (*n* = 4), *ts2/* *+* *;Ts5/* *+* (*n* = 5), + */* *+* *; Ts5/−* (*n* = 8), and wild type *(* *+* */−;* *+* */* *+* ) (*n* = 6)) are shown. Boxes for genetic classes with completely feminized tassels are orange. These classes were not significantly different (ns) by one-way ANOVA with Tukey’s multiple comparison test, *P* < 0.05. For *ts2;* *+* */* *+* vs *ts2/* *+* *; Ts5*, *q* = 3.26787, multiplicity-adjusted *P* = 0.3376, 95% CI = −0.105368 to 0.575774, for *ts2*; + / + vs. ts2; *Ts5/* *+* , *q* = 1.60441, multiplicity adjusted *P* = 0.9314, 95% CI = −0.456047 to 0.225095, for *ts2*; + / + vs. ts2; *Ts5*, *q* = 0.825390, multiplicity adjusted *P* = 0.9977, 95% CI = −0.473261 to 0.332678. Dashes represent pooled classes wherein the allele may be mutant or wild type at the locus. Bars, mean; whiskers, SD. **b** Unpollinated ears and mature tassels of *sk1;Ts5* double mutants in the Mo17 background. White arrowheads indicate elongated ear shanks. **c** A model for the actions of known gene products of the maize JA pathway.

Given the down-regulation of *ts2* in *Ts5* mutants, we made a double mutant to assay the potential dosage effects between the mutant loci. The crosses were carried out in A188, which is a moderately expressive background for both mutations. *ts2* is known to be completely recessive, and its genetic interaction with *Ts5* in a heterogeneous genetic background has been previously described^8^. Spikes were completely feminized in plants homozygous for *ts2*, independent of *Ts5* dosage (Fig. 4a). Plants with an FSL/SL ratio of 0 are males and those with a ratio of 1 are completely feminized. Surprisingly, lowering the dose of TS2 increased the feminization of *Ts5* tassels. *Ts5*/ + ; + / + ; and *Ts5/Ts5*; + / + (*n* = 8) plants had variable FSL/SL ratios with a mean of less than 0.20 while double heterozygotes, *Ts5*/ + ; *ts2*/ + ; (*n* = 5), had a mean ratio of almost 0.60 (Fig. 4a). These results suggest different possible routes for a feminization switch. That switch can be reached by mutating *ts2* or overexpressing *Ts5*, or by a combination of both.

We also crossed *Ts5* to *silkless1* (*sk1*), which is in the same genetic pathway with *ts2* and *ts1*^8,42,43^*. sk1* is a uridine diphosphate glycosyltransferase that blocks the production of JA^44^. *Ts5;sk1* double homozygous mutants in the Mo17 background were silkless in the ear and had just a few silks formed in the tassel, indicating that *sk1* is epistatic (Fig. 4b). Because *Ts5* functions later in the pathway to catabolize JA-Ile and SK1 functions earlier to block the production of JA-Ile, we reason that sufficient JA-Ile is still present to prevent silks in both the tassel and ear of the double mutant.

Interestingly, we observed elongated ear shanks in both *Ts5* and double mutants in this family (Fig. 4b). This phenotype was described in *opr7opr8* mutants and is seen in *teosinte branch1* (*tb1*) mutants^45,46^, a gene which is downregulated in *Ts5* (Supplementary Data 2). The elongated internodes of the ear shank contrast with the shortened internodes below the terminal inflorescence. Perhaps this differential response is due to the differences in JA metabolism or signaling in these two organs, or results from the differences in the amounts of any other hormone.

## Discussion

Maize plants have separate male and female flowers (are monecious) due to pistil abortion in the tassel and stamen arrest in the ear. In addition, maize ears contain straight rows of kernels due to the abortion of the lower floret. In *Ts5*, pistil abortion is blocked with the result that the tassel is feminized and kernel rows in the ear are irregular. These phenotypes are shared by *ts1, ts2*, and *opr7opr8*, as well as by lines that overexpress *SK1*. TS1 is a lipoxygenase needed for the biosynthesis of JA, and TS2 is a short-chain alcohol dehydrogenase purported to be in the JA pathway^9,11^. The study of its substrate specificity, however, suggests that it more likely affects steroid compounds such as brassinosteroids and may not play a direct role in the jasmonate signaling pathway^47^. SK encodes a uridine diphosphate glycosyltransferase that blocks the production of JA^44^. Thus, JA biosynthesis and metabolism are critical for pistil abortion in the tassel and abortion of the lower floret in the ear.

Here, we present evidence that the dominant *Ts5* mutant is due to the misexpression of a maize homolog of *Arabidopsis, CYP94Bs*. In *Arabidopsis*, CYP94B1^26^ and CYP94B3^27^ catabolize JA-Ile, the active jasmonate metabolite that binds to the nuclear localized jasmonate co-receptor complex^32^. Transcript levels of *ZmCYP94B1* are dramatically upregulated in *Ts5* mutant tassels, and jasmonate metabolites are altered consistently with the increased catabolism of JA-Ile through the ω-oxidation pathway^25^. *Ts5* leaves have ~ 4-fold more *ZmCYP94B1* transcripts prior to wounding, and transcript levels continue to rise over time, failing to return to non-wounded levels even a day after wounding. Indeed, the catabolic products of JA-Ile remain high in wounded *Ts5* leaves, unlike those of wild types. The increase in *ZmCYP94B1* transcripts in *Ts5* tassel and leaf, along with changes in JA metabolism, support the hypothesis that the feminization of *Ts5* tassels is due to reduced JA-Ile levels, similar to the observations of *ts1* or *opr7opr8*^9,14^.

The genetic lesion of *Ts5* is unknown. The first mention of *Ts5* is on a genetic map in 1932 without further description^21^. Given the novel 5′ transcripts found by RNA-Seq and the reduction in recombination around the locus, it is likely to be a genomic rearrangement. Reduced recombination can be found in heterozygous genomic regions of differing retrotransposon haplotypes^48^. Our mapping population, made by backcrossing a *Ts5*/+ parent in an unknown progenitor background to Mo17, may indeed have a structural variation that affects genetic distances by contracting or expanding physical and genetic distances between markers^48^. Moreover, we were unable to amplify the 5′-end of the gene using PCR, possibly due to the presence of a large insertion such as a transposon. Unique dominant alleles often result from large rearrangements that alter *cis* regulatory sequences, such as *Abphyl2*, which is a transposition^49^, *Kn1-O*, which is a tandem duplication^50^ or *Wab1-R*, which is ectopically expressed in the leaf due to unknown *cis* regulatory changes^51^.

If higher jasmonate levels lead to pistil abortion in the tassel, what keeps it from eliminating the pistils in the ear? This quandary was first investigated in 1925 in the analysis of *silkless1* (*sk1*)^42,52^. Tassels of *sk1* mutants are normal, but ears lack silks, although they retain other female features such as stamen suppression and short glumes. The double mutant with *ts2* restores the silks to the ear, while the tassel remains mostly staminate^8,43^. Although the exact substrate of SK1 is unknown, its overexpression leads to a *tasselseed* phenotype and reduces 12-oxo-phytodienoic acid (OPDA, a precursor of JA) and JA-Ile^44^. The authors hypothesize that SK1 inactivates JA or a precursor in a tissue-specific manner. The rescue of silks in the *sk1;ts2* double mutant occurs because the loss of SK1 has no effect in the *ts2* mutant background^43^. *SK1* is expressed at very low levels, but is most highly expressed in ears, consistent with its function to protect the silks of ears. The *Ts5;sk1* double mutant is different from *ts2;sk1* in that no silks form in the ear and very few in the tassel, plus lower floret abortion still occurs in ears. The absence of SK1 is expected to cause an increase in JA and, consequently, its downstream product JA-Ile. Overexpression of *ZmCYP94B1* in *Ts5* catabolizes JA-Ile, but the turnover effect may be somewhat compensated by the higher levels of JA-Ile. This result is similar to the rescue of *Ts5* mutants by exogenous JA. The increased catabolism from *Ts5* is unable to compensate for the extra jasmonate predicted in the *sk1* mutant.

Although sex determination in maize is dependent upon jasmonate suppression of pistils, regulating pistil outgrowth does not explain why tasselseed florets have pistils and no stamens. The function of jasmonate in promoting stamen development is well established in *Arabidopsis*. Mutants that are deficient in jasmonate biosynthesis, perception, or signaling are also defective in stamen differentiation^12,32,53^. In fact, the overexpression of CYP94B1 or CYP94B3 in *Arabidopsis* leads to a partial loss of fertility. The stigma is extended, the anther filament is shortened, and pollen viability is reduced^27^. We also saw a similar phenotype when overexpressing Ts5 in *Arabidopsis*. The high levels of JA and JA-Ile in wild-type tassels compared to ears and the repression of stamens in JA maize mutants suggest that jasmonate is needed in both maize and *Arabidopsis* for proper stamen development.

Additional jasmonate-deficient phenotypes are described in rice that are consistent with sex-determination phenotypes in maize. The allene oxide cyclase mutants of rice have elongated sterile lemmas and, occasionally, longer palea or additional bract-like organs^47^ similar to tassel glumes. The *eg1* mutant, which encodes a plastid-targeted lipase, a homolog of DEFECTIVE IN ANTHER DEHISCENCE1 (DAD1) of *Arabidopsis*, and the *eg2* mutant, defective in JAZMONATE ZIM-DOMAIN (JAZ) gene, have extra glume-like organs and altered floral organ numbers^54^. In *Sorghum*, pedicellate spikelets are normally sterile but not in *multiseeded (msd)* mutants that produce extra spikelets. The increase in fertile spikelets in *msd1* mutants is lost with the addition of JA^55^. Clearly, jasmonate plays a role in blocking the growth of pistils, leaf-life organs, and spikelets in addition to its well-established role in stamen fertility.

Our analysis of Ts5 adds to the growing understanding of how jasmonates are involved in maize sex determination and that not only its biosynthesis but also its turnover plays a role by contributing to its homeostasis (Fig. 4c). The lipoxygenase TS1 putatively converts α-linolenic acid (18:3) to an intermediary and then to *cis*-( + )-12-oxophytodienoic acid (OPDA)^9^, a precursor to JA-Ile that is further metabolized to JA via the function of OPR7 and OPR8, which are partially and functionally redundant^14^. The activation of the short-chain dehydrogenase/reductase TS2 requires TS1^43^. Although, its exact substrates are unknown^47^, TS2 promotes JA production. Once the bioactive jasmonate, JA-Ile, is made, it relieves transcription factors from JAZ repression^32^, leading to the activation of several pathways, including those necessary for floral development^12,32,53^. *ZmCYP94B1* then acts to inactivate JA-Ile by oxidation.

In conclusion, study of the gain-of-function mutant, *Ts5*, has revealed that the proper expression of *ZmCYP94B1* is necessary to maintain maize monoecy. The conversion of JA-Ile to 12OH-JA-Ile via the *ZmCYP94B1* enzyme is an important regulatory mechanism for sex determination in normal ear and tassel development. Given that this enzyme also has a role in ameliorating wound-induced JA response in leaves, it provides an elegant example of a gene product having multiple tissue-specific functions.

## Methods

### Plant materials and growth conditions

The *Ts5-ref* allele was obtained from the Maize Cooperative Center and backcrossed at least seven times to B73 and Mo17 and four times to A188. Mapping was performed after backcrossing seven times into Mo17 population. The *ts2-ref* and *ts1-ref* alleles were obtained from the Maize Cooperative Center. Genetic analysis of *Ts5;ts2* double mutants was performed in the A188 background after three backcrosses and genotyping of all possible alleles. Introgressed stocks of *sk1-R* in Mo17 were a gift from Erik Vollbrecht (Iowa State). Genetic analysis of *Ts5;sk1* double mutants was performed after crossing *sk1-R* in Mo17 to *Ts5* in Mo17 and self-pollinating the F1 to create an F2 population. Ecotype Col-0 was used for all *Arabidopsis thaliana* experiments, and plants were grown under standard greenhouse conditions. Primers CL 682 and CL 758 (Supplementary Data 1) were used to amplify *Ts5* (GRMZM2G177668), which has no introns, from B73 genomic DNA. The resulting amplicon was cloned into the pENTR/D-TOPO Vector using the manufacturer’s instructions (Thermo Fisher Scientific) to make a Gateway compatible entry vector. pEarleyGate103^56^ was linearized with *Eco*RV and recombined with the *Ts5* entry clone via LR Clonase™ II Reaction. The resulting clone places the *Ts5* ORF under control of the *cauliflower mosaic virus* 35S promoter. Plants were transformed using the floral dip method^57^. Transformants were also genotyped and transgene expression was confirmed with primers CL 682 and CL 758 (Supplementary Data 1). *CYP94B3-OE* lines have been described^27^.

### Plant treatments

Plants for the rescue of tassel feminization were treated with JA as in Yan et al.^14^.

For the root-growth assay, plants were grown on filter paper in the dark at 30 °C for 5 days or grown in the medium vermiculite for 10 days under normal greenhouse conditions.

### Genetic mapping of the *Ts5* locus

We fine mapped *Ts5* to a 15 Mb interval flanked by custom indel markers CL589.590 and MS13.14 in a backcross population of *Ts5* to Mo17 consisting of 273 plants. This was further narrowed to the interval flanked by umc2039 and TIDP9218. DNA extraction was performed using standard protocols (Lunde, 2018)^58^ using the primers listed in Supplementary Data 1.

### RNA-expression analysis

RNA-Seq libraries were constructed as in Tsuda et al.^59^ except that 6 µg of total RNA was used rather than 3 µg. Three libraries were made of four pooled 9–11 mm tassels: two mutants and one wild type. Libraries were sequenced on a NextSeq Illumina platform with 75 bp paired-end reads. Raw reads were aligned to the maize B73 genome AGPv3.30 using HISAT2^60^ and counted to AGPv3.30 gene models using HTSeq-counts^61^ using the cyberinfrastructure provided by Cyverse Atmosphere^62^. Reads were visualized using the Integrative Genomics Viewer (Broad Institute)^63^. Counted reads were tested for differential expression with edgeR using a generalized linear model (GLM) approach on transcripts with a raw count greater than 5 in at least one condition and FDR significance threshold of 0.05^64,65^. Differentially expressed genes (FDR ≤ 0.05) between Ts5 and WT siblings were separated by -log(fold-change) into upregulated and downregulated differentially expressed gene lists. Gene accessions from each list were tested for Gene Ontology term enrichment by singular Gene Ontology term enrichment analysis (SEA) with agriGO v2.0^66^. Quantitative reverse transcription PCR was performed as in Bolduc et al.^67^.

### Phylogenetic analysis of maize CYP94 genes

The amino-acid sequence of GRMZM2G177668_P01 was blasted against *Zea mays* AGPv3.30 and against *A. thaliana* Araport11 genomes^68–70^. Canonical protein isoforms with blastp bit scores > 100 were run in an ETE3^71^ pipeline that included alignment by Clustal Omega^72^ phylogeny model evaluation using PhyML^73^ and tree branchpoint evaluation using 100 bootstraps. Trees were visualized and annotated in R^74^ with the ggtree package^75^.

### Analytical methods and chemicals

Hormone extraction from tissues was according to a previously described method with minor modifications^31^ for the quantification of jasmonate by mass spectrometry. Frozen leaf tissues and inflorescences (100‒200 mg) were pulverized using metal beads in TissueLyser II (Qiagen) and extracted multiple times with 3 ml of ethyl acetate (0.5% acetic acid) containing a mixture of dihydro-JA (dhJA) and [^13^C_6_]-JA-Ile as an internal standard. The combined extracts were evaporated under a stream of nitrogen gas, and the dried residue was reconstituted in 0.2 ml of 70% methanol/water/acetic acid (v/v/v = 70:29.5:0.5). The resulting tissue extract was cleared by centrifugation at 18,000 × *g* for 30 min in 4 °C. Analysis and quantification of jasmonate were carried out based on methods described previously^26^ using an electrospray ionization triple quadrupole mass spectrometer (Xevo T-QS, Waters) interfaced with an ultra-performance liquid chromatography (ACUITY H-class, Waters). Characteristic mass spectrometry transitions detected under multiple reaction monitoring in electrospray ionization-negative mode were for JA (*m/z* 209 > 59), dhJA (211 > 59), 12OH-JA (225 > 59), JA-Ile (322 > 130), [^13^C_6_]-JA-Ile (328 > 136), 12OH-JA-Ile (338 > 130), and 12COOH-JA-Ile (352 > 130). MassLynx 4.1 and TargetLynx (Waters) were used to analyze the data.

### Statistical analysis and plotting

Student’s *t* tests, one-way analysis of variance, and graphs were made using GraphPad Prism software^76^, with the exception of graphs in 3F, which were designed in core R packages^74^. Raw data used to create graphs are available in Supplementary Data 4.

### Reporting Summary

Further information on experimental design is available in the Nature Research Reporting Summary linked to this article.

## Supplementary information

Supplementary Information

Description of Supplementary Data

Supplementary Data 1

Supplementary Data 2

Supplementary Data 3

Supplementary Data 4

Reporting Summary

## Data Availability

Transcriptomic data for 10 mm tassel RNA-Seq of Ts5/ + and normal siblings are available at the NCBI sequence read archive (SRA) under the accession code PRJNA495059.
