# Supplementary material for: Tasselseed5 overexpresses a wound-inducible enzyme, ZmCYP94B1, that affects jasmonate catabolism, sex determination, and plant architecture in maize
Source: Commun Biol. 2019 Mar 25;2:114. doi: 10.1038/s42003-019-0354-1 (PMC6433927; doi:10.1038/s42003-019-0354-1)
Supplement: Supplementary file 1 — Supplementary Information [file 42003_2019_354_MOESM1_ESM.docx]

**
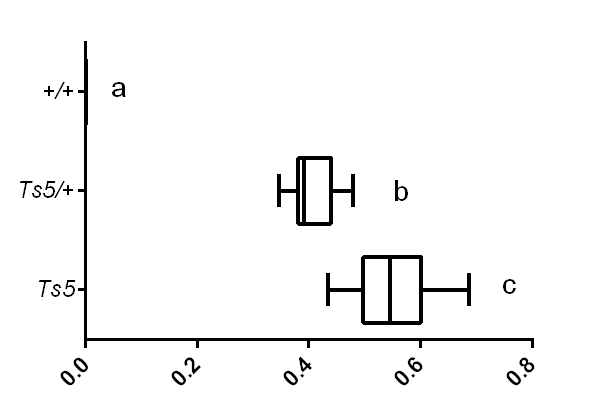
**

**Supplementary Figure 1. Dosage of *Ts5* allele affects tassel feminization (FSL/SL).** Heterozygotes (*Ts5*/+) have intermediate feminization phenotype (FSL/SL) when observed in A188. Plant sample sizes are as follows: +/+ n=8, *Ts5*/+ n=11 and *Ts5* n=13. All 3 groups are significantly distinct by one-way ANOVA, using an uncorrected Fisher’s LSD, DF=29, all comparisons have *P* < 0.0001. For *Ts5/+* vs. *+/+*, *t*=16.87757, for *Ts5* vs. *Ts5/+*, *t*=6.548853, for *Ts5* vs. *+/+*, *t*=23.42278.


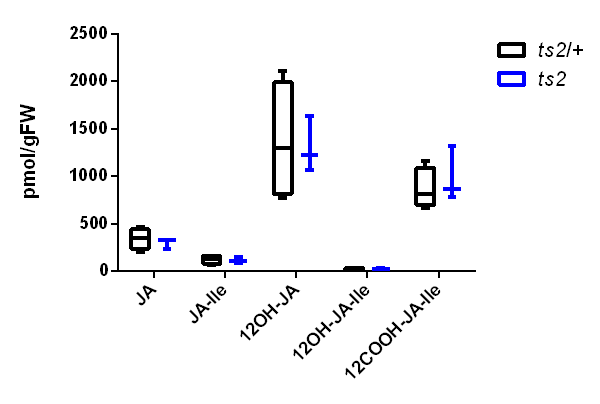


**Supplemental Figure 2. *ts2* mutant leaf jasmonate levels are unchanged 4 hours post-wounding**. Graph depicting of LC-MS outputs of JA, JA-Ile, 12OH-JA, 12OH-JA-Ile, and 12COOH-JA-Ile accumulation (pmol/gFW) in *ts2* (n=3 biological replicates) and *ts2/+* (n=4 biological replicates), in A188, at 4 hours post wounding. Bars are set at the mean. Differences between the means were not significant, two-tailed Student’s *t*-test at p>0.05.
