# Supplementary material for: Tasselseed5 overexpresses a wound-inducible enzyme, ZmCYP94B1, that affects jasmonate catabolism, sex determination, and plant architecture in maize
Source: Commun Biol. 2019 Mar 25;2:114. doi: 10.1038/s42003-019-0354-1 (PMC6433927; doi:10.1038/s42003-019-0354-1)
Supplement: Supplementary file 2 — Description of Supplementary Data [file 42003_2019_354_MOESM2_ESM.docx]

**Description of Supplementary Data**

**Filename: Supplementary Data 1**

**Description:** List of primers used in this study. Names of primers are in the first column followed by sequences listed 5’ to 3’.

**Filename: Supplementary Data 2**

**Description:** **Complete list of DEGs in *Ts5/+* developing tassels**. Differentially-expressed genes (FDR <= 0.05) between *Ts5/+* and WT siblings were separated by log(fold-change) into up-regulated and down-regulated differentially-expressed gene lists.

**Filename: Supplementary Data 3**

**Description: Enriched GO information in DEGs of *Ts5/+* developing tassels.** Singular gene ontology (GO) term enrichment analysis within down-regulated and up-regulated differentially expressed genes between Ts5 and WT siblings, listing the GO term identifier, ontological category (P = biological process, F = molecular function), GO term description, visual representation of enrichment, enrichment FDR statistic, and number of gene models with that ontology found in the test set. Heat colors correspond to the level of enrichment, where red is most significantly enriched and yellow is least significantly enriched. Grey GO terms are not significantly enriched.

**Filename: Supplementary Data 4**

**Description:** Raw data tables for graphs in Figures 1 to 4 and in Supplementary Figure 1 and Supplementary Figure 2.
