# Supplementary material for: Tasselseed5 overexpresses a wound-inducible enzyme, ZmCYP94B1, that affects jasmonate catabolism, sex determination, and plant architecture in maize
Source: Commun Biol. 2019 Mar 25;2:114. doi: 10.1038/s42003-019-0354-1 (PMC6433927; doi:10.1038/s42003-019-0354-1)
Supplement: Supplementary file 3 — Supplementary Data 1 [file 42003_2019_354_MOESM3_ESM.docx]

**Supplemental Data 1.** Primers used in this study

| Primer/ Oligo | 5’→3 |
| --- | --- |
| **Mapping Primers** |  |
| CL 589 | TGCTGCTCTATGACGCATCT |
| CL 590 | GCCAAGCATTTTGTTTCGAT |
| umc2039F | CATCTCCTACCAGCTCACCCC |
| umc2039R | GCTCGGGGTAGTAGTGTTCTCCTT |
| JW35 | AGAGGCCTCGTCTCCCTAAG |
| JW36 | ATCACCTTGCTCACCGTCTC |
| IDP454F | GACGCAACTAAGCAGCTCG |
| IDP454R | GCTCTATGGTGAATGTCTGGC |
| TIDP4643F | GATGAAGACGAGCCACTTCC |
| TIDP4643R | TGGATATGGAGACCCTCTGG |
| mmc0471F | TTAGCACATTTGAAGAGTTTTG |
| mmc0471R | TTTCCTTCACGTTTCTCTGT |
| TIDP9218F | ACCAACAACCTCGGTACTGC |
| TIDP9218R | TGCTGGCATCTTCTACTCCC |
| MS13 | CTAGGTCTTCGCTGGGTCC |
| MS14 | TGTCTCTTCCTACTGCCTGC |
|  |  |
| **RT-PCR Primers** |  |
| ZmGAPDHF | CCTGCTTCTCATGGATGGTT |
| ZmGAPDHR | TGGTAGCAGGAAGGGAAACA |
| Ts5 qPCRF (CL 679) | ATGGCCTTCGTGCAGATGAA |
| Ts5 qPCRR (CL 675) | GGATCGAGTGGAGTAGCAGC |
|  |  |
| **Genotyping primers** |  |
| Ts5 in Mo17 (umc2039F) | CATCTCCTACCAGCTCACCCC |
| Ts5 in Mo17 (umc2039R) | GCTCGGGGTAGTAGTGTTCTCCTT |
| Ts5 in B73F (CL 674) | ACACGCAATGTTTTTGCTGC |
| Ts5 in B73R (CL 658) | GGCCGTATCTTCGCTGGATA |
| ts2 (phi001F) | TGACGGACGTGGATCGCTTCAC |
| ts2 (phi001R) | AGCAGGCAGCAGGTCAGCAGCG |

Names of custom primers are in the first column followed by sequences listed 5’ to 3’.
