# Supplementary material for: Tasselseed5 overexpresses a wound-inducible enzyme, ZmCYP94B1, that affects jasmonate catabolism, sex determination, and plant architecture in maize
Source: Commun Biol. 2019 Mar 25;2:114. doi: 10.1038/s42003-019-0354-1 (PMC6433927; doi:10.1038/s42003-019-0354-1)
Supplement: Supplementary file 6 — Supplementary Data 4 [file 42003_2019_354_MOESM6_ESM.docx]

**Supplementary Data 4. Raw data tables for graphs in main text figures 1 to 4 and supplementary information figures 1 and 2.**

Table of data for Figure 1H, heights, including tassels (cm).

| N | *Ts5/+* |
| --- | --- |
| 196.3 | 165.0 |
| 188.5 | 180.2 |
| 190.8 | 175.6 |
| 181.4 | 179.7 |
| 207.9 | 179.4 |
| 188.9 | 174.5 |
| 189.4 | 180.9 |
| 198.6 | 190.0 |
| 178.3 | 181.9 |
| 182.2 | 178.9 |
| 188.9 | 182.2 |
| 206.1 | 176.1 |
| 180.8 | 172.9 |
| 186.4 | 164.6 |
| 197.0 | 170.9 |
| 188.8 | 172.4 |
| 184.7 | 160.1 |
| 212.5 | 181.8 |
| 201.8 | 170.2 |
| 209.5 | 192.4 |
| 197.5 | 192.4 |
| 203.3 | 170.1 |
| 194.5 | 179.4 |
| 195.0 | 192.8 |
| 184.5 | 167.1 |
| 200.4 | 174.4 |
| 187.3 | 178.2 |
| 189.9 | 185.7 |
| 200.0 | 188.1 |
| 207.6 | 180.2 |
|  | 191.0 |

Table of data for Figure 1I – Internode lengths (cm)

| 6^th^ Ts5 | 6^th^ B73 | 5^th^ *Ts5* | 4^th^ | 4^th^ B73 | 3^rd^ *Ts5* | 3^rd^ B73 | 2^nd^ *Ts5* | 2^nd^ B73 | 1^st^ *Ts5* | 1^st^ B73 |
| --- | --- | --- | --- | --- | --- | --- | --- | --- | --- | --- |
| 14.5 | 11.5 | 18.0 | 14.5 | 18.5 | 14.0 | 18.5 | 14.0 | 18.0 | 16.0 | 25.0 |
| 13.0 | 13.0 | 17.0 | 17.0 | 17.5 | 17.0 | 18.0 | 17.0 | 18.5 | 17.0 | 24.0 |
| 13.5 | 14.0 | 16.5 | 16.5 | 18.5 | 16.0 | 18.5 | 16.5 | 20.0 | 16.5 | 26.5 |
| 14.0 | 13.5 | 18.5 | 17.5 | 18.5 | 17.0 | 16.5 | 17.5 | 17.0 | 16.5 | 21.5 |
| 16.0 | 14.0 | 18.5 | 17.5 | 18.0 | 17.5 | 17.5 | 17.0 | 17.5 | 17.5 | 21.0 |
| 14.0 | 12.5 | 18.0 | 16.0 | 19.5 | 15.5 | 19.5 | 15.5 | 20.0 | 16.0 | 22.5 |
| 14.0 | 14.0 | 17.5 | 17.0 | 19.5 | 17.0 | 20.5 | 16.0 | 20.5 | 16.5 | 26.0 |
| 14.0 | 15.0 | 17.5 | 18.0 | 17.0 | 18.0 | 18.0 | 16.5 | 18.5 | 16.5 | 25.5 |
| 13.5 | 14.5 | 16.5 | 15.5 | 17.5 | 15.5 | 17.5 | 17.0 | 18.0 | 16.0 | 23.5 |
| 13.5 | 13.0 | 16.0 | 13.5 | 18.5 | 16.5 | 18.5 | 16.5 | 16.0 | 16.5 | 20.5 |
| 14.5 | 17.5 | 17.0 | 17.5 | 16.5 | 16.5 | 16.5 | 15.5 | 17.0 | 15.5 | 20.0 |
| 18.0 | 14.5 | 18.5 | 16.5 | 18.0 | 15.5 | 16.5 | 15.0 | 15.0 | 15.0 | 14.0 |
| 11.5 | 15.0 | 17.5 | 16.5 | 18.5 | 14.5 | 19.0 | 15.0 | 18.5 | 15.0 | 22.5 |
| 15.0 | 11.0 | 15.5 | 14.5 | 16.5 | 15.5 | 17.5 | 17.0 | 17.5 | 17.5 | 22.0 |
| 16.5 | 13.5 | 18.0 | 14.0 | 17.5 | 15.0 | 17.0 | 16.5 | 17.0 | 15.0 | 24.0 |
| 13.0 | 13.5 | 18.0 | 16.5 | 18.0 | 15.5 | 17.0 | 16.0 | 16.5 | 15.5 | 23.0 |
| nd | 12.0 | 14.5 | 15.0 | 18.5 | 16.0 | 19.0 | 15.5 | 18.5 | 15.5 | 26.5 |
| 15.5 | 14.0 | 19.0 | 17.0 | 18.5 | 16.5 | 18.0 | 16.0 | 19.0 | 17.0 | 25.0 |
| 14.5 | 14.5 | 18.5 | 16.0 | 18.0 | 17.0 | 18.0 | 17.0 | 16.0 | 17.0 | 19.0 |
| 17.5 | 15.0 | 16.5 | 18.0 | 18.0 | 18.0 | 17.5 | 18.0 | 17.0 | 18.0 | 21.0 |
| 14.0 | 16.0 | 17.5 | 17.0 | 18.0 | 17.0 | 18.0 | 15.5 | 18.5 | 15.0 | 20.0 |
| 14.5 | 13.0 | 17.5 | 15.5 | 18.5 | 16.0 | 18.0 | 15.5 | 17.5 | 18.0 | 22.5 |
| 17.0 | 13.0 | 18.0 | 16.5 | 18.0 | 15.5 | 18.0 | 15.5 | 18.5 | 16.0 | 22.5 |
| 13.0 | 14.0 | 16.0 | 16.5 | 18.0 | 16.5 | 18.5 | 16.5 | 19.0 | 16.0 | 25.5 |
| 13.5 | 15.0 | 17.0 | 16.0 | 18.5 | 16.0 | 18.0 | 15.5 | 18.5 | 15.5 | 21.5 |
| 16.0 | 15.5 | 19.0 | 16.5 | 18.5 | 16.5 | 18.0 | 16.5 | 17.0 | 16.0 | 19.0 |
| 17.0 | 14.5 | 17.5 | 16.5 | 17.5 | 16.0 | 18.0 | 15.5 | 16.5 | 16.5 | 15.5 |
| 13.5 | 13.5 | 16.0 | 17.5 | 18.0 | 17.5 | 18.5 | 17.5 | 19.5 | 18.5 | 23.0 |
| 17.0 | 13.0 | 18.5 | 17.0 | 19.5 | 16.0 | 18.0 | 14.0 | 17.0 | 17.0 | 22.5 |
| 14.0 | 15.0 | 19.0 | 15.5 | 18.5 | 16.5 | 18.0 | 16.5 | 17.5 | 13.5 | 23.5 |
|  | 14.5 |  | 17.0 |  | 18.5 |  | 20.0 |  | 21.0 |  |
|  |  |  |  |  |  |  |  |  |  |  |

Table of data for Figure 2C

| *ts1* | *ts1*+JA | *Ts5* | *Ts5*+JA | *ts1* | *ts1*+JA | *Ts5* | *Ts5*+JA |
| --- | --- | --- | --- | --- | --- | --- | --- |
| 1.000000 | 0. | 1.000000 | 0.0 | 0.2857143 | 0.000000 | 0.5490196 | 0.000000 |
| 1.000000 | 0. | 1.000000 | 0.0 | 0.3666667 | 0.2857143 | 0.3913043 | 0.000000 |
| 1.000000 | 0. | 1.000000 | 0.0 | 0.187500 | 0.000000 | 0.3939394 | 0.000000 |
| 0.9302326 | 0. | 0.900000 | 0.0 | 0.000000 | 0.000000 | 0.6136364 | 0.000000 |
| 0.6818182 | 0. | 1.000000 | 0.0 | 0.000000 | 0.000000 | 0.3939394 | 0.000000 |
| 0.7391304 | 0. | 0.900000 | 0.5 | 0.000000 | 0.000000 | 0.6136364 | 0.7704918 |
| 0.9310345 | 0. | 0.8571429 | 0.0 | 0.000000 | 0.000000 | 0.5238096 | 0.000000 |
| 0.7419355 | 0. | 0.875000 | 0.0 | 0.000000 | 0.000000 | 0.509434 | 0.000000 |
| 0.8484849 |  | 0.9444444 | 0.0 | 0.000000 |  | 0.500000 | 0.000000 |
| 1.000000 |  | 1.000000 | 0.0 | 0.5333334 |  | 0.5510204 | 0.000000 |
| 1.000000 |  | 0.950000 | 0.0 | 0.4583333 |  | 0.600000 | 0.000000 |
|  |  | 1.000000 |  |  |  | 0.4782609 |  |

Table of data for Figure 2D

| A188 | *ts1* | *Ts5* | B73 |
| --- | --- | --- | --- |
| 22.2 | 48.0 | 31.0 | 12.4 |
| 12.7 | 42.5 | 25.2 | 3.0 |
| 10.0 | 46.5 | 29.7 | 11.0 |
| 40.1 | 23.5 | 0.0 | 5.2 |
| 0.0 | 21.0 | 29.5 | 16.0 |
| 15.5 | 63.0 | 30.0 | 2.0 |
| 0.0 | 14.7 | 10.0 | 12.1 |
| 55.7 | 21.5 | 0.0 | 2.1 |
| 14.0 | 24.0 | 29.3 | 10.3 |
| 10.2 | 36.9 | 26.0 | 4.5 |
|  | 18.7 | 30.7 | 17.0 |
|  | 35.5 | 17.0 |  |

Table of data for Figure 2E

| WT | 35S::CYP94B3 | 35S::ZmCYP94B1 |
| --- | --- | --- |
| 0.000000 | 0.2207792 | 0.7446808 |
| 0.01694915 | 0.2118644 | 0.9272727 |
| 0.000000 | 0.1323529 | 0.351145 |
| 0.000000 | 0.3513514 | 0.8917198 |
| 0.009090909 | 0.7101449 | 0.2596154 |

Table of data for Figure 3C

| JA B73 | JA-Ile B73 | 12OH-JA-Ile B73 | 12COOH-JA-Ile B73 | JA Ts5 | JA-Ile Ts5 | 12OH-JA-Ile Ts5 | 12COOH-JA-Ile Ts5 |
| --- | --- | --- | --- | --- | --- | --- | --- |
| 3729.2130 | 798.695300 | 42.84233 | 91.90207 | 1522.1610 | 387.427200 | 297.40240 | 139.54580 |
| 3911.9590 | 686.759700 | 33.11861 | 75.49907 | 2027.0470 | 552.257200 | 330.70190 | 112.85740 |
| 1709.1880 | 283.932600 | 31.41085 | 73.48715 | 1415.5920 | 330.675800 | 710.73190 | 356.93030 |
| 3980.1850 | 936.044200 | 63.93830 | 84.59857 | 3133.1650 | 981.900200 | 472.11040 | 251.91320 |

Table of data for Figure 3D

| JA 1 | 2 | 3 | JA-Ile 1 | 2 | 3 |
| --- | --- | --- | --- | --- | --- |
| 61.37172 | 56.109620 | 3.242032 | 30.71296 | 12.025960 | 0.220657 |
| 45.10679 | 10.141650 | 37.263090 | 22.69713 | 2.987339 | 1.870500 |
| 85.86170 | 4.418483 | 27.182260 | 27.51809 | 1.260646 | 3.427529 |
|  | 42.256760 | 33.612040 |  | 22.700660 | 10.234230 |
|  | 29.462970 |  |  | 13.412350 |  |

Table of data for Figure 3E

| REP1 | Sample | CtGAPDH | CtTs5 |  |  | DCt(Ts5-GAPDH) | DDCt(Ts5-GAPDH) |
| --- | --- | --- | --- | --- | --- | --- | --- |
|  | B73 0HR | 21.90 | 24.72 | B73 |  | 2.82 | 0.00 |
|  | B73 2HR | 23.54 | 24.20 |  |  | 0.66 | -2.16 |
|  | B73 24HR | 22.82 | 24.92 |  |  | 2.10 | -0.72 |
|  | Ts5 0HR | 22.31 | 25.46 | Ts5 |  | 3.16 | 0.34 |
|  | Ts5 2HR | 22.82 | 24.33 |  |  | 1.51 | -1.31 |
|  | Ts5 24HR | 23.40 | 24.53 |  |  | 1.13 | -1.69 |
| REP2 |  |  |  |  |  |  |  |
|  | B73 0HR | 22.31 | 24.95 | B73 |  | 2.64 | 0.00 |
|  | B73 2HR | 22.77 | 24.39 |  |  | 1.62 | -1.02 |
|  | B73 24HR | 23.24 | 25.04 |  |  | 1.80 | -0.84 |
|  | Ts5 0HR | 22.22 | 25.82 | Ts5 |  | 3.60 | 0.96 |
|  | Ts5 2HR | 22.84 | 24.48 |  |  | 1.64 | -1.00 |
|  | Ts5 24HR | 23.81 | 24.01 |  |  | 0.20 | -2.44 |
| REP3 |  |  |  |  |  |  |  |
|  | B73 0HR | 22.24 | 25.07 | B73 |  | 2.82 | 0.00 |
|  | B73 2HR | 23.24 | 24.42 |  |  | 1.18 | -1.65 |
|  | B73 24HR | 23.43 | 24.73 |  |  | 1.30 | -1.53 |
|  | Ts5 0HR | 22.77 | 25.40 | Ts5 |  | 2.63 | -0.19 |
|  | Ts5 2HR | 23.17 | 24.25 |  |  | 1.08 | -1.75 |
|  | Ts5 24HR | 24.37 | 24.29 |  |  | -0.08 | -2.90 |

Table of data for Figure 3F

|  | pmol/gFW |  |  |  |  |  |
| --- | --- | --- | --- | --- | --- | --- |
|  | JA | JA-Ile | 12OH-JA | 12OH-JA-Ile | 12COOH-JA-Ile |  |
| Ts5/Ts5 (T0) | 20.63622015 | 0.551729 | 1170.049 | 1.85462492 | 21.54802219 |  |
| Ts5/Ts5 (T0) | 14.08489173 | 0.169053 | 543.9489 | 0.86203182 | 11.23642893 |  |
| Ts5/Ts5 (T0) | 13.8195052 | -0.09442 | 581.2503 | 0.91804301 | 23.63814937 |  |
| Ts5/Ts5 (T0) | 3.72498168 | 0.073176 | 459.8708 | 0.49548342 | 9.622442461 |  |
| Ts5/+ (T0) | 1.217225911 | 0.147788 | 366.5387 | 1.24578558 | 10.0971164 |  |
| Ts5/+ (T0) | 7.777906519 | -0.00279 | 504.2937 | 1.01422072 | 8.966068205 |  |
| Ts5/+ (T0) | 5.031428307 | 0.048762 | 410.5269 | 0.74127638 | 9.368084682 |  |
| Ts5/+ (T0) | 2.363250984 | 0.024039 | 278.9297 | 0.38012647 | 5.248704717 |  |
| B73 (T0) | 13.64449283 | 0.089598 | 569.8385 | 1.04623718 | 7.261262838 |  |
| B73 (T0) | 21.57919937 | 0.011723 | 340.4015 | 1.05881373 | 6.24132953 |  |
| B73 (T0) | 13.21949051 | 0.390007 | 450.9551 | 0.83954413 | 6.955557181 |  |
| B73 (T0) | 8.245110596 | 0.062428 | 421.5233 | 0.31775183 | 2.076982581 |  |
| Ts5/Ts5 (T1) | 89.45945639 | 30.96829 | 711.8751 | 7.42357713 | 25.89854531 |  |
| Ts5/Ts5 (T1) | 138.1443557 | 40.80416 | 1106.138 | 15.8082749 | 48.17726057 |  |
| Ts5/Ts5 (T1) | 100.0263942 | 29.82047 | 593.1813 | 7.57266745 | 22.9332541 |  |
| Ts5/Ts5 (T1) | 79.12958718 | 21.95144 | 487.5469 | 2.3753426 | 8.564366767 |  |
| Ts5/+ (T1) | 188.6395077 | 73.15879 | 1074.342 | 1.86758817 | 7.520127227 |  |
| Ts5/+ (T1) | 130.1148862 | 40.73858 | 487.7761 | 3.05075152 | 7.031082955 |  |
| Ts5/+ (T1) | 152.9728104 | 68.65396 | 598.9401 | 3.18092365 | 8.361071449 |  |
| Ts5/+ (T1) | 214.3157647 | 107.9905 | 806.1369 | 7.60331799 | 15.88439088 |  |
| B73 (T1) | 161.7915541 | 54.55086 | 479.6108 | 1.64731729 | 3.972810874 |  |
| B73 (T1) | 232.8143937 | 83.78242 | 678.1332 | 4.42051717 | 6.039998918 |  |
| B73 (T1) | 226.372485 | 83.98316 | 565.2964 | 4.90956966 | 12.30572765 |  |
| B73 (T1) | 275.8812908 | 101.0786 | 894.3016 | 6.60667709 | 13.02984972 |  |
| Ts5/Ts5 (T4) | 33.92264325 | 8.966839 | 814.5677 | 4.1049767 | 60.9464861 | *outlier |
| Ts5/Ts5 (T4) | 192.3415824 | 119.718 | 1724.36 | 70.6990407 | 648.9344641 |  |
| Ts5/Ts5 (T4) | 135.7653347 | 80.72984 | 1657.377 | 39.9755196 | 347.0949722 |  |
| Ts5/Ts5 (T4) | 190.1532568 | 112.3339 | 1733.699 | 57.0548599 | 394.9025941 |  |
| Ts5/+ (T4) | 41.59918815 | 19.31322 | 548.01 | 1.82761781 | 25.00753784 | *outlier |
| Ts5/+ (T4) | 144.2738478 | 56.7282 | 1060.269 | 19.7229892 | 419.3958994 |  |
| Ts5/+ (T4) | 153.3566811 | 33.80513 | 1325.639 | 13.8269405 | 225.9589736 |  |
| Ts5/+ (T4) | 159.0665972 | 118.1779 | 1867.725 | 51.563639 | 690.0298178 |  |
| B73 (T4) | 20.55488843 | 9.452692 | 276.6135 | -0.31447506 | 16.49854767 | *outlier |
| B73 (T4) | 258.0578267 | 137.9071 | 1379.202 | 27.9915667 | 376.4966966 |  |
| B73 (T4) | 298.1511411 | 159.8471 | 920.943 | 50.5851457 | 651.7909312 |  |
| B73 (T4) | 236.7772627 | 108.7124 | 1158.378 | 24.1397509 | 332.1503652 |  |
| Ts5/Ts5 (T6) | 85.16516069 | 46.9625 | 1038.883 | 28.4855113 | 561.4616823 | *outlier |
| Ts5/Ts5 (T6) | 138.0983071 | 77.52243 | 2083.806 | 55.1807129 | 878.3897198 |  |
| Ts5/Ts5 (T6) | 140.0328178 | 75.06984 | 1691.056 | 47.9950449 | 832.8786409 |  |
| Ts5/Ts5 (T6) | 97.58298165 | 66.32237 | 1749.764 | 48.7065063 | 677.4623556 |  |
| Ts5/+ (T6) | 132.8821582 | 104.6955 | 1413.571 | 45.3820029 | 561.6273344 |  |
| Ts5/+ (T6) | 140.1316148 | 93.71552 | 1287.538 | 41.4396803 | 609.8301592 |  |
| Ts5/+ (T6) | 138.7880621 | 117.8527 | 1554.544 | 50.7385374 | 598.584607 |  |
| Ts5/+ (T6) | 183.8817866 | 115.2596 | 1853.032 | 48.1416066 | 549.2130285 |  |
| B73 (T6) | 127.3476474 | 97.56821 | 1338.479 | 21.7813099 | 493.634081 |  |
| B73 (T6) | 173.630235 | 75.26425 | 1245.658 | 16.5430882 | 334.7850344 |  |
| B73 (T6) | 155.6931379 | 58.53577 | 1027.972 | 18.8942285 | 317.1655658 |  |
| B73 (T6) | 262.652017 | 109.3787 | 1729.908 | 40.1242497 | 748.0504706 | *outlier |
| Ts5/Ts5 (T24) | 39.86993225 | 32.84741 | 1007.352 | 23.375374 | 884.0962759 |  |
| Ts5/Ts5 (T24) | 38.33443752 | 42.42146 | 1842.008 | 32.9122703 | 1248.648039 |  |
| Ts5/Ts5 (T24) | 30.0764222 | 33.73988 | 1677.461 | 33.0947785 | 1255.611674 |  |
| Ts5/Ts5 (T24) | 42.50829162 | 23.377 | 797.112 | 15.7694803 | 743.6838747 | *outlier |
| Ts5/+ (T24) | 56.26720706 | 23.5009 | 1168.295 | 5.58276884 | 265.0904914 |  |
| Ts5/+ (T24) | 23.46872158 | 34.75381 | 749.0118 | 12.7370764 | 727.2661801 |  |
| Ts5/+ (T24) | 52.3993498 | 35.92382 | 1045.844 | 14.9456096 | 640.1979416 |  |
| Ts5/+ (T24) | 45.24489447 | 33.6453 | 652.2388 | 9.62900026 | 522.5368906 |  |
| B73 (T24) | 38.0470588 | 15.20951 | 719.5759 | 2.41400339 | 208.5749439 |  |
| B73 (T24) | 70.14849615 | 36.17117 | 657.7253 | 4.17641559 | 239.1512581 |  |
| B73 (T24) | 44.25357389 | 24.6257 | 742.7276 | 4.72754086 | 233.5479005 |  |
| B73 (T24) | 63.94764054 | 25.36314 | 615.2128 | 3.71669184 | 264.8320916 |  |

Table of data for Figure 4A

| *+/-; +/+* | *+/+; Ts5/-* | *ts2/+; Ts5/+* | *ts2; +/+* | *ts2/+; Ts5* | *ts2; Ts5/+* | *ts2; Ts5* |
| --- | --- | --- | --- | --- | --- | --- |
|  |  |  |  |  |  |  |
| .000000 | 0.000000 | 0.5535714 | 0.6538461 | 0.547619 | 0.9166667 | 0.7555556 |
| 0.000000 | 0.08333334 | 0.5849057 | 0.8333333 | 0.5217391 | 0.8888889 | 0.9534883 |
| 0.000000 | 0.000000 | 0.520000 | 0.880000 | 0.5365854 | 1.000000 | 0.9574468 |
| 0.000000 | 0.326087 | 0.4230769 | 0.9069768 | 0.6603774 | 0.8461539 |  |
| 0.000000 | 0.9183673 | 0.5416667 |  | 0.6086956 | 1.000000 |  |
| 0.000000 | 0.000000 |  |  |  | 0.952381 |  |
|  | 0.050000 |  |  |  |  |  |
|  | 0.000000 |  |  |  |  |  |

Table of data for Supplementary Information Figure 1

| *Ts5* | *Ts5/+* | *+/+* |
| --- | --- | --- |
| 0.5217391 | 0.3913043 | 0. |
| 0.4761905 | 0.4347826 | 0. |
| 0.5909091 | 0.3809524 | 0. |
| 0.440000 | 0.4090909 | 0. |
| 0.5263158 | 0.3913043 | 0. |
| 0.687500 | 0.3461538 | 0. |
| 0.5714286 | 0.375000 | 0. |
| 0.5185185 | 0.4545455 | 0. |
| 0.5909091 | 0.440000 |  |
| 0.6111111 | 0.4782609 |  |
| 0.6111111 | 0.3913043 |  |
| 0.4347826 |  |  |
| 0.5454545 |  |  |

Table of data for Supplementary Information Figure 2

|  | ts2/+ |  |  |  | ts2/ts2 |  |  |  |
| --- | --- | --- | --- | --- | --- | --- | --- | --- |
|  | Bio Rep1 | Rep2 | Rep3 | Rep4 | Bio Rep1 | Rep2 | Rep3 | Rep4 |
| JA | 206.880 | 321.530 | 380.850 | 461.520 | nd | 330.60 | 326.07 | 231.90 |
| JA-Ile | 68.450 | 119.230 | 162.900 | 146.630 | nd | 84.67 | 148.96 | 108.34 |
| 12OH-JA | 769.650 | 947.820 | 2105.380 | 1647.400 | nd | 1636.12 | 1226.11 | 1068.01 |
| 12OH-JA-Ile | 14.460 | 27.520 | 32.900 | 35.670 | nd | 26.44 | 24.31 | 38.60 |
| 12COOH-JA-Ile | 666.290 | 836.050 | 782.480 | 1166.270 | nd | 868.24 | 785.61 | 1318.15 |
