# Supplementary material for: Tasselseed5 overexpresses a wound-inducible enzyme, ZmCYP94B1, that affects jasmonate catabolism, sex determination, and plant architecture in maize
Source: Commun Biol. 2019 Mar 25;2:114. doi: 10.1038/s42003-019-0354-1 (PMC6433927; doi:10.1038/s42003-019-0354-1)
Supplement: Supplementary file 7 — Reporting Summary [file 42003_2019_354_MOESM7_ESM.pdf]

nature research

Corresponding author(s): Dr. Sarah Hake

Reporting Summary

Nature Research wishes to improve the reproducibility of the work that we publish. This form provides structure for consistency and transparency in reporting. For further information on Nature Research policies, see Authors & Referees and the Editorial Policy Checklist.

Statistical parameters

When statistical analyses are reported, confirm that the following items are present in the relevant location (e.g. figure legend, table legend, main text, or Methods section).

n/a	Confirmed	
☐	☒	The exact sample size (n) for each experimental group/condition, given as a discrete number and unit of measurement
☐	☒	An indication of whether measurements were taken from distinct samples or whether the same sample was measured repeatedly
☐	☒	The statistical test(s) used AND whether they are one- or two-sided<br>*Only common tests should be described solely by name; describe more complex techniques in the Methods section.*
☒	☐	A description of all covariates tested
☐	☒	A description of any assumptions or corrections, such as tests of normality and adjustment for multiple comparisons
☐	☒	A full description of the statistics including central tendency (e.g. means) or other basic estimates (e.g. regression coefficient) AND variation (e.g. standard deviation) or associated estimates of uncertainty (e.g. confidence intervals)
☐	☒	For null hypothesis testing, the test statistic (e.g. F, t, r) with confidence intervals, effect sizes, degrees of freedom and P value noted<br>*Give P values as exact values whenever suitable.*
☒	☐	For Bayesian analysis, information on the choice of priors and Markov chain Monte Carlo settings
☒	☐	For hierarchical and complex designs, identification of the appropriate level for tests and full reporting of outcomes
☒	☐	Estimates of effect sizes (e.g. Cohen's d, Pearson's r), indicating how they were calculated
☐	☒	Clearly defined error bars<br>*State explicitly what error bars represent (e.g. SD, SE, CI)*

Our web collection on statistics for biologists may be useful.

Software and code

Policy information about availability of computer code

Data collection	No computer code was used for data collection.

Data analysis	Raw reads were aligned to the maize B73 genome AGPv3.30 using HISAT2 (Kim et al. 2015), and counted to AGPv3.30 gene models using HTSeq-counts (Anders et al. 2015) using the cyberinfrastructure provided by Cyverse Atmosphere (Goff et al. 2011). Reads were visualized using the Integrative Genomics Viewer (Broad Institute) (Thorvaldsdóttir et al. 2013). Counted reads were tested for differential expression with edgeR using a GLM approach on transcripts with a raw count greater than 5 in at least one condition, and FDR significance threshold of 0.05 (Robinson et al. 2010; McCarthy et al. 2012). Differentially-expressed genes (FDR <= 0.05) between Ts5 and WT siblings were separated by -log(fold-change) into up-regulated and down-regulated differentially-expressed gene lists. Gene accessions from each list were tested for GO term enrichment by singular GO term enrichment analysis (SEA) with agriGO v2.0 (Tian et al. 2017).The amino acid sequence of GRMZM2G177668_P01 was blasted against Zea mays AGPv3.30 and against Arabidopsis thaliana Araport11 genomes (Cheng et al. 2017; Cannon et al. 2011; Berardini et al. 2015). Canonical protein isoforms with blastp bit scores >100 were run in an ETE3 (Huerta-Cepas et al. 2016) pipeline that included alignment by Clustal Omega (Sievers et al. 2014) phylogeny model evaluation using PhyML (Guindon et al. 2010) and tree branchpoint evaluation using 100 bootstraps. Trees were visualized and annotated in R (R Foundation for Statistical Computing 2018) with the ggtree package (Yu et al. 2017). Student's t-tests, one-way ANOVA and graphs in Fig.1H, 2C, 4A, S1 and S2 or described in the text were made using GraphPad Prism software (Miller 2003). All others were made using Microsoft Excel 2016. MassLynx 4.1 and TargetLynx (Waters) were used to analyze the LC-MS data. No custom algorithms were used in this study.

For manuscripts utilizing custom algorithms or software that are central to the research but not yet described in published literature, software must be made available to editors/reviewers upon request. We strongly encourage code deposition in a community repository (e.g. GitHub). See the Nature Research guidelines for submitting code & software for further information.

Data

Policy information about availability of data

All manuscripts must include a data availability statement. This statement should provide the following information, where applicable:

- Accession codes, unique identifiers, or web links for publicly available datasets
- A list of figures that have associated raw data
- A description of any restrictions on data availability

Sequence data that supports this study will be available in the SRA of Genbank. All other data that support the findings of this study are available from the corresponding author upon reasonable request.

Field-specific reporting

Please select the best fit for your research. If you are not sure, read the appropriate sections before making your selection.

☒ Life sciences ☐ Behavioural & social sciences ☐ Ecological, evolutionary & environmental sciences

For a reference copy of the document with all sections, see nature.com/authors/policies/ReportingSummary-flat.pdf

Life sciences study design

All studies must disclose on these points even when the disclosure is negative.

Sample size	A sample size of 30 plants was attempted for vigorous genotypes. For genotypes of low vigor or fertility or low occurrence a minimum sample size of 5 was used because this was within possible resource constraints and allowed for detection of difference between sample statistics. Pools of 4 tassels were used in the RNA-Seq experiment because after dissection of 96 plants, only 12 were at the necessary size/ developmental stage for our study.
Data exclusions	not applicable
Replication	Four biological replicates were used for LC-MS experiments. Four biological and 3 technical replicates were used for quantitative RT-PCR experiments.
Randomization	not applicable
Blinding	not applicable

Reporting for specific materials, systems and methods

Materials & experimental systems

n/a	Involved in the study
☐	☒ Unique biological materials
☒	☐ Antibodies
☒	☐ Eukaryotic cell lines
☒	☐ Palaeontology
☒	☐ Animals and other organisms
☒	☐ Human research participants

Methods

n/a	Involved in the study
☒	☐ ChIP-seq
☒	☐ Flow cytometry
☒	☐ MRI-based neuroimaging

Unique biological materials

Policy information about availability of materials

Obtaining unique materials	Maize genetic stocks of reference mutant alleles described in this study are available from the Maize Genetics Cooperation Stock Center. All other described genetic stocks are freely available from the authors.
